# Three-dimensional (3D) cell culture: a valuable step in advancing treatments for human hepatocellular carcinoma

**DOI:** 10.1186/s12935-022-02662-3

**Published:** 2022-07-30

**Authors:** Asmaa F. Khafaga, Shaker A. Mousa, Lotfi Aleya, Mohamed M. Abdel-Daim

**Affiliations:** 1 Department of Pathology, Faculty of Veterinary Medicine, Alexandria University, Edfina, 22758 Egypt; 2 Pharmaceutical Research Institute, Albany College of Pharmacy and Health Sciences, Rensselaer, NY 12144 USA; 3 Chrono-Environnement Laboratory, UMR CNRS 6249, Bourgogne Franche-Comté University, 25030 Besançon Cedex, France; 4 Pharmacology Department, Faculty of Veterinary Medicine, Suez Canal University, Ismailia, 41522 Egypt; 5 Present Address: Department of Pharmaceutical Sciences, Pharmacy Program, Batterjee Medical College, P.O. Box 6231, Jeddah 21442, Saudi Arabia

**Keywords:** HCC, 3D cell culture, TME, Drug resistance, 2D cell culture, Chemotherapeutic drugs

## Abstract

Hepatocellular carcinoma (HCC) is the fifth most common malignant cancer and the third most frequent cause of tumour-related mortality worldwide. Currently, several surgical and medical therapeutic strategies are available for HCCs; however, the interaction between neoplastic cells and non-neoplastic stromal cells within the tumour microenvironment (TME) results in strong therapeutic resistance of HCCs to conventional treatment. Therefore, the development of novel treatments is urgently needed to improve the survival of patients with HCC. The first step in developing efficient chemotherapeutic drugs is the establishment of an appropriate system for studying complex tumour culture and microenvironment interactions. Three-dimensional (3D) culture model might be a crucial bridge between in vivo and in vitro due to its ability to mimic the naturally complicated in vivo TME compared to conventional two-dimensional (2D) cultures. In this review, we shed light on various established 3D culture models of HCC and their role in the investigation of tumour-TME interactions and HCC-related therapeutic resistance.

**Graphical Abstract:**

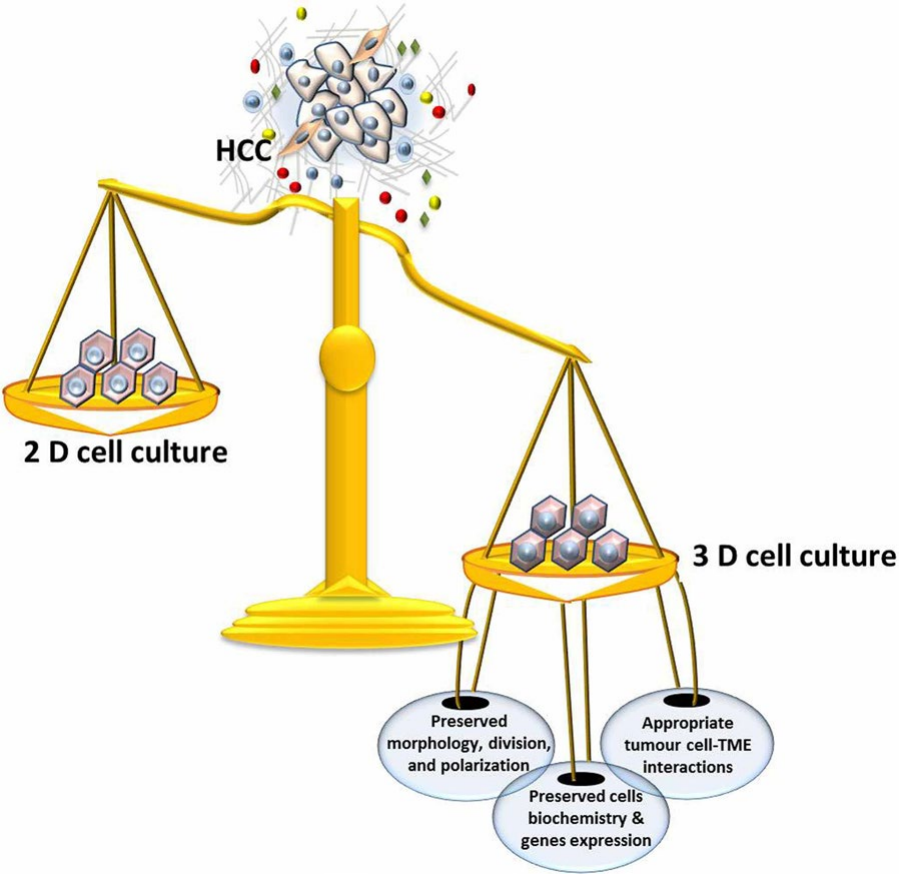

## Introduction

Hepatocellular carcinoma (HCC) is the third leading cause of cancer-related death worldwide [1–3] and commonly has a poor prognosis. In particular, patients with HCC usually develop severe chronic hepatic damage, including inflammatory, fibrotic, and cirrhotic lesions. Thus, these patients frequently show poor systemic chemotherapeutic tolerance [4]. Currently, efficient strategies for treating HCCs are still lacking due to the continuous growth of tumour cells and the genetic complexity involving molecular factors that affect interactions between tumour cells and their surrounding tumour microenvironment (TME) [5–7].

Investigating the complex features of neoplastic cells and the tumour-TME interaction has proven to be a consistent challenge. Therefore, the production of optimal in vivo and in vitro systems involved in the complex neoplastic and non-neoplastic cell interactions requires more effort [8]. Most of the available data regarding in vitro systems are derived from two-dimensional (2D) culture systems, which are simple, inexpensive, cell-based models. However, these methods cannot replicate the specific architecture and biochemical signalling of cells in vivo. Hence, switching from a 2D system to a three-dimensional (3D) system is necessary to better understand HCC tumour biology [9, 10] Developing appropriate 3D models that better mimic the specificity of the TME has received great scientific interest, and there were more than 1000 publications on this topic in 2016 alone [8]. The current review provides an overview of the different 3D culture models of HCC and their role in investigating tumour-TME interactions as well as HCC-related therapeutic resistance. Topics covered in this review include the therapeutic resistance of HCC to conventional treatment, TME factors affecting HCC progression, differences between 2 and 3D culture models, and the development of novel models of HCC 3D culture spheroids. Our aim is to provide a comprehensive review to expand the knowledge in the field of HCC treatment strategies.

## HCC and resistance to conventional therapeutics

**Fig. 1:**
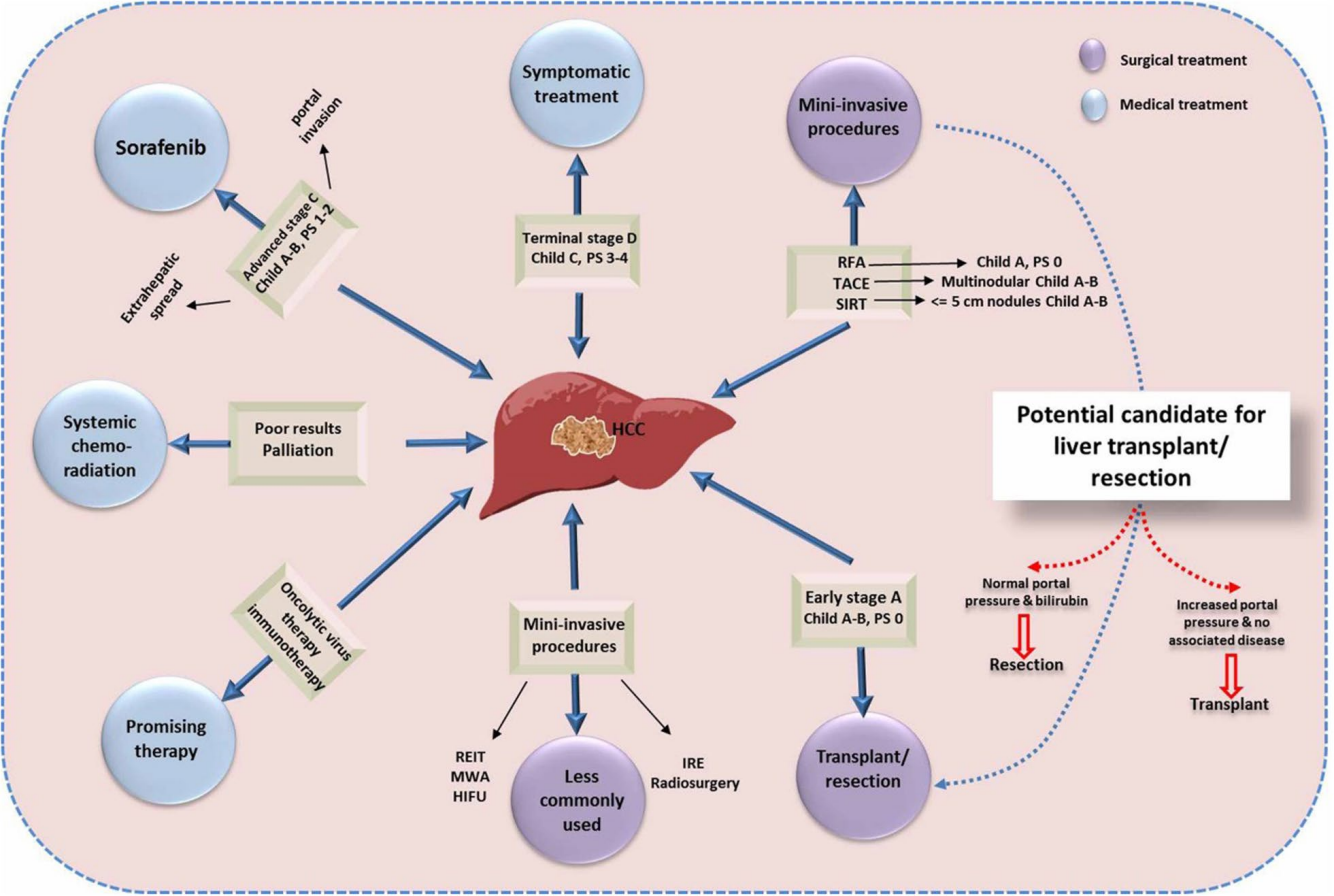
The role of tumour staging in guiding the treatment decisions according to the Child–Pugh classification

HCC is well established as the most common form of primary liver malignancy, the fifth most common type of overall malignant cancerglobally [11–13]**.** Currently, several modalities, including liver transplantation, surgical intervention, radiology, systemic chemotherapy, and trans-arterial chemoembolization, are available for the treatment of patients with late-stage HCC [14]. However, the cure rate of patients with HCC is very low [15–17], and the recurrence rate of HCC after surgical resection is usually high due to tumour metastasis and a reduced efficacy of conventional chemotherapy [13, 18, 19] The low survival rateof patients with late stage HCC could be due tp the lack of highly reliable biomarkers to identify the early stage cancer, besides the presence of primary underlying liver dysfunction, which limits the efficacy of available radio and chemotherapy like sorafenib [20, 21]. Sorafenib, the multi-kinase inhibitor that is the only Food and Drug Administration (FDA)-approved molecular targeted drug for advanced-stage HCC [22, 23], provides about three months survival on average [24]. Additionally, HCC is known for its poor response and increased intrinsic resistance for both local regional and systemic cytotoxic chemotherapy [22]**.** Several factors could control the tumor resistance including abnormal TME, activation of epithelial-mesenchymal transition (EMT), heterogeneity of HCC, and induction of various signalling pathways [25]. Among them, TME is the most widely investigated factor due to its role in tumor progression and chemotherapeutic resistance [26, 27]. Therefore, evaluation of the potential anticancer efficacy of novel drugs commonly targets the TME as a marker for therapeutic competence against HCC [28, 29]. The role of tumour staging according to the Child–Pugh classification in guiding treatment decisions is summarized in Fig. 1.

## TME factors affect HCC progression and therapeutic resistance

**Fig. 2:**
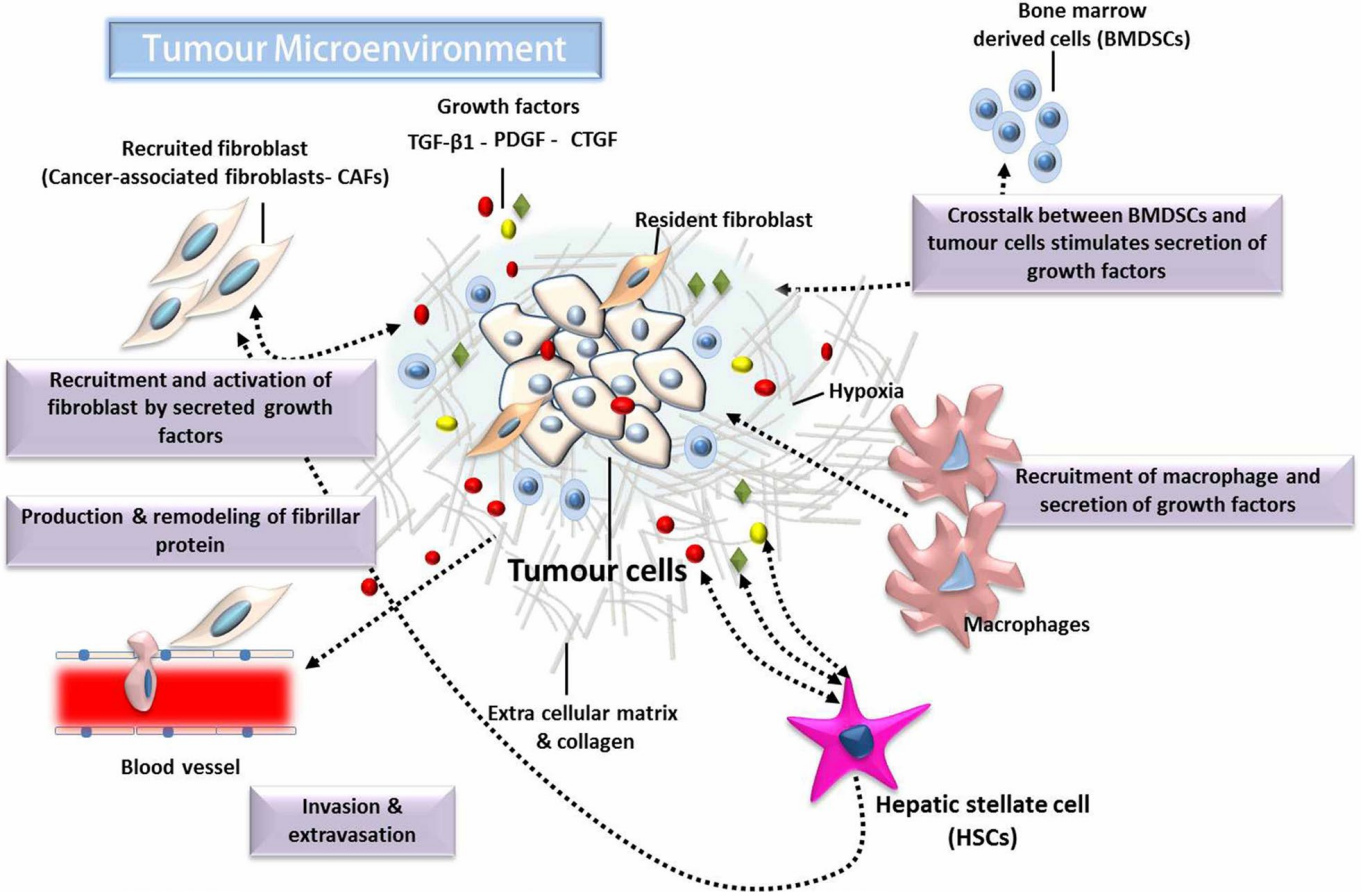
The cellular components comprise the HCC microenvironment. *TGF-β1* Transforming growth factor beta, *PDGF* Platelet-derived growth factor, *CTGF* Connective tissue growth factor

The TME comprises various non-neoplastic cells that contribute to multiple aspects of disease progression, including growth, metastasis, migration, and the development of chemotherapeutic resistance [8]. As represented in Fig. 2, the major components of the HCC microenvironment are malignant tumour cells and various non-cancerous stromal cells. Cancer-associated fibroblasts (CAFs) and hepatic stellate cells (HSCs) are the main cells within the HCC stroma, in addition to endothelial, immune, and inflammatory cells [30]. All these cell types play specific roles in promoting tumour structure and function [31].

CAFs could be produced from several cell types, including resident and bone marrow-derived fibroblasts, and they play a pivotal role in the promotion of HCC growth and metastasis via the production of various growth factors and cytokines [32]. However, the major production site of activated CAFs is HSCs [33, 34]. HSCs could flood the TME with increased amounts of different types of growth factors, such as connective tissue growth factor (CTGF), transforming growth factor β1 (TGF-β1), and platelet-derived growth factor (PDGF). These factors are able to activate HCC cells and HSCs through both paracrine and autocrine pathways [35, 36]. Bidirectional activation of the TME consequently resulted in enhanced extracellular matrix (ECM) synthesis, tumour proliferation and invasion, as well as increased therapeutic resistance [37, 38].

It is now strongly suggested that the continuous changes in the histopathologic sequences of different tumours might result from the interaction between cancer cells and the surrounding non-neoplastic cells during tumorigenesis, which indicates the importance of investigating the TME [39]. Therefore, targeting HCC-HSCs and the HCC-CAF interaction became crucial in the investigation of supressing HCC growth [36, 40]. To better understand such interactions, the use of appropriate models for tumour cells and microenvironment culture is essential. Hence, the development of gradational 3D cancer models, which may largely mimic the interaction between TME and cancer cells, is crucial to investigate the mechanisms of tumour progression and promising novel chemotherapeutic drugs.

## The superiority of 3d cell culture over traditional 2D cell culture

The pathogenesis and potential therapeutic agents for HCC have been extensively studied in various animal models. However, the presence of several factors limited the efficacy of those models, including increased cost, long implementation, and difficulty in obtaining human fibroblasts [41]. In addition, certain experimental conditions may result in the development of many questions about the animals’ pain and discomfort. As such, the immune systems of animal models may be compromised during the experiment with subsequent alterations of tumour cell-TME interaction (unlike in humans), which limits the clinical application of novel investigations [42]. Obtaining a convenient link between clinical trials and experimental animal models is an everyday challenge [43].

2D cell cultures are a well-known, attractive laboratory method used for simple assays that probe cell physiology and behaviour. In 2D cultures, the cell culture monolayer grows on polystyrene or glass materials in a simple environment that does not realistically recapitulate in vivo tissue physiology. Therefore, the resultant cells usually have modifications in their architecture, polarity, biochemical signalling, and, importantly, cell–cell interactions [9]. Additionally, in 2D tumour cell cultures, several major features of cancer cells, including heterogeneity as well as morphological and genetic profiles, are mostly lost [8]. Hence, switching from a 2D system to a 3D system may provide a better simulation of the complex biology of malignant tumours for study. An ideal 3D culture would overcome 2D culture limitations and allow direct drug applications in human models by conserving the original cell morphology, polarity, heterogeneity, and genetic profile of both neoplastic and non-neoplastic stromal cells [8, 44]. Although there are great advantages of 3D culture versus 2D culture, the simplicity and low cost of 2D systems, as well as the lack of a universal model for a 3D system, has limited the use of 3D cultures. Currently, several 3D culture models have been developed for liver, pancreatic, and breast cancer to clarify the role of the tumour-TME interaction in drug resistance [45–48]. Interestingly, 3D tumour homo-spheroids were observed to exhibit increased resistance to tested chemotherapy compared to the responses in 2D cancer models [49–51]. The advantages and disadvantages of 3D and 2D cell culture systems are summarized in Table 1.

**Table 1:** Advantages and disadvantages of three dimensional (3D) and two dimensional (2D) cell culture systems

Items		2D cell culture	3D cell culture	Refs.
Disadvantages	Time required for culture formation	Minutes to a few hours	A few hours to a few days	[52]
	Quality of culture	Simple long-term culture Easy to interpret results High performance and reproducibility	More difficult to culture Difficult to interpret results Poor performance and reproducibility	[53]
	Cost of culture maintenance	Less time consuming Inexpensive Commercially available media and assay materials	More time consuming More expensive Fewer commercially available assay materials	[44, 45]
Advantages	In vivo imitation	Cannot mimic the natural tumour mass structure	Can mimic in vivo tissue structures	[55]
	Cell interactions	No cell–cell or cell- extracellular microenvironment interactions No “niches” or in vivo-like microenvironment	Appropriate cell–cell and cell-extracellular microenvironment interactions Microenvironment “niches” are present	[56–58]
	CellCharacteristics	Altered morphology from physiological tissue Altered cell division activity Lack of diverse phenotypes and polarization	Preserved morphology Preserved cell division activity Presence of diverse phenotypes and polarization	[59, 60]
	Access to essential compounds	Limited access to nutrients, oxygen, metabolites, and signalling molecules	Variable access to nutrients, oxygen, metabolites, and signalling molecules	[61, 62]
	Molecular mechanisms	Alterations in cellular biochemistry Alterations in gene expression, mRNA splicing, and topology	Preserved cellular biochemistry Preserved gene expression, mRNA splicing, and topology	[63, 64]
	Angiogenesis	Only observational	Could be functional	[65]
	Mathematical model	Possible	Better geometry and structure–function links	[65]

## Biomaterial-based 3D liver models

The construction of three-dimensional (3D) tissue models in vitro is critical for drug discovery research and development. The use of biomaterials to improve the design of cell function and activity is critical in this regard. Despite the fact that the 2D and 3D systems were utilized for different purposes, the 3D culture is preferable in terms of drug development because it closely resembles the in vivo cancer environment. The active use of biomaterials is another way for improving cell functioning [66].

Cell culture is frequently carried out on a dish or plate made primarily of polystyrene. Because the artificial environment differs significantly from the in vivo body environment of cancer cells, evaluating therapeutic impact or cytotoxicity is technologically constrained. Biomaterials containing extracellular matrix (ECM) components have been shown to improve cell activity and function. Cells will be able to improve their proliferation, differentiation, and biological capabilities as a result of their interaction with biomaterials, resulting in the realisation of cancer cell–environment interaction. Several studies have been published on 3D cancer models integrated with biomaterials to reproduce the cancer environment and illnesses in vitro [67–70].

Natural biomaterials are sourced from animals or plants, while synthetic biomaterials are created artificially. Polysaccharide (amylose, cellulose, alginate, chitosan, or hyaluronic acid), peptide (collagen or gelatin), nucleic acid, or polyhydroxyalkanoates are all examples of natural biomaterials. Most natural biomaterials can be destroyed enzymatically since the degradative enzyme and metabolic system already exist in the body [71]. Natural biomaterials are frequently employed to construct the 3D culture system of cancer cells because the components that make up the cancer environment, such as the ECM, contribute to cancer diseases. Although natural biomaterials are highly biocompatible, they do have some immunogenicity and homogeneity limits. Synthetic biomaterials are employed to avoid the problems. The majority of synthetic biomaterials are destroyed nonenzymatically through simple hydrolysis. Synthetic biomaterials have several advantages, such as the ability to control characteristics, high rigidity, and property clarity [66].

Several 3D cancer cell culture systems including biomaterials are presented. In the 3D culturing system of cancer cells, two types of biomaterials have been used so far. One is a cancer cell culturing system using biomaterials in a spherical shape. Cancer cells spontaneously form a cell aggregate with a tissue-like 3D structure when incubated with microspheric hydrogels of biomaterial, which simulates the cancer environment [72]. The difficulty of separating cells from cell-hydrogel complexes is one of the system's drawbacks, and as a result, the results are sometimes inconsistent. The other is a cancer cell culture method using non-spherical biomaterials such as sponge forms or nonwoven textiles. Cells effectively multiply and move on the scaffold in this system; this form is appropriate for immunohistochemistry [66].

Basement membrane extract (BME/Matrigel) is a well-defined soluble basement membrane extract that derived from an epithelial tumor [73]. It has a composition similar to real basement membrane and forms a hydrogel at 24–37 °C [74]. It's utilized in vitro as a 3D cell culture substrate, in suspension for spheroid culture, and for a variety of tests, including angiogenesis, invasion, and dormancy. However in vivo*,* BME/Matrigel is used for angiogenesis experiments and to promote the take and growth of xenograft and patient-derived biopsy samples [75]. According to several studies, the rigidity of the BME/Matrigel and its components are both responsible for its activity with so many distinct cell types. BME/Matrigel is widely used in assays and models to help the better understanding of tumor biology and develop treatment methods [73].

Individual tumor types can be better modeled with BME/Matrigel. For example, new stiffer and acidic matrices including extra ECM proteins are holding promise for more physiologically relevant models because they better simulate tumor settings. Coculture in vitro and in vivo has advanced the establishment of a physiological tumor microenvironment significantly [73, 76]. Many malignancies are fibrotic, and the use of fibroblasts has helped us better understand how these cells interact with tumors [77]. Cancer stem cells have emerged as a key 'actor' in the tumor field, with BME/Matrigel-based assays assisting in defining their identity, biology, and involvement in malignancy. When BME/Matrigel was first characterized in 1986 as a "basement membrane complex with biological activity," it seemed to play a key function in cell differentiation [73]. Multiple uses in cancer biology, such as 3D culture, invasion assays, endothelial tube tests, dormancy assays, multicellular tumor spheroids, xenografts, and patient-derived xenografts, were not anticipated [78].

In terms of tumor metastasis, coculture of tumor cells with endothelial cells has revealed some unexpected interactions for some tumor types with vasculature [79]. The use of a BME/Matrigel based assay to better understand the genes and processes of this type of metastatic spread along the outside of vasculature and nerves will begin to address the selection of therapeutic methods.

## Development of novel 3D models for HCC

**Fig. 3:**
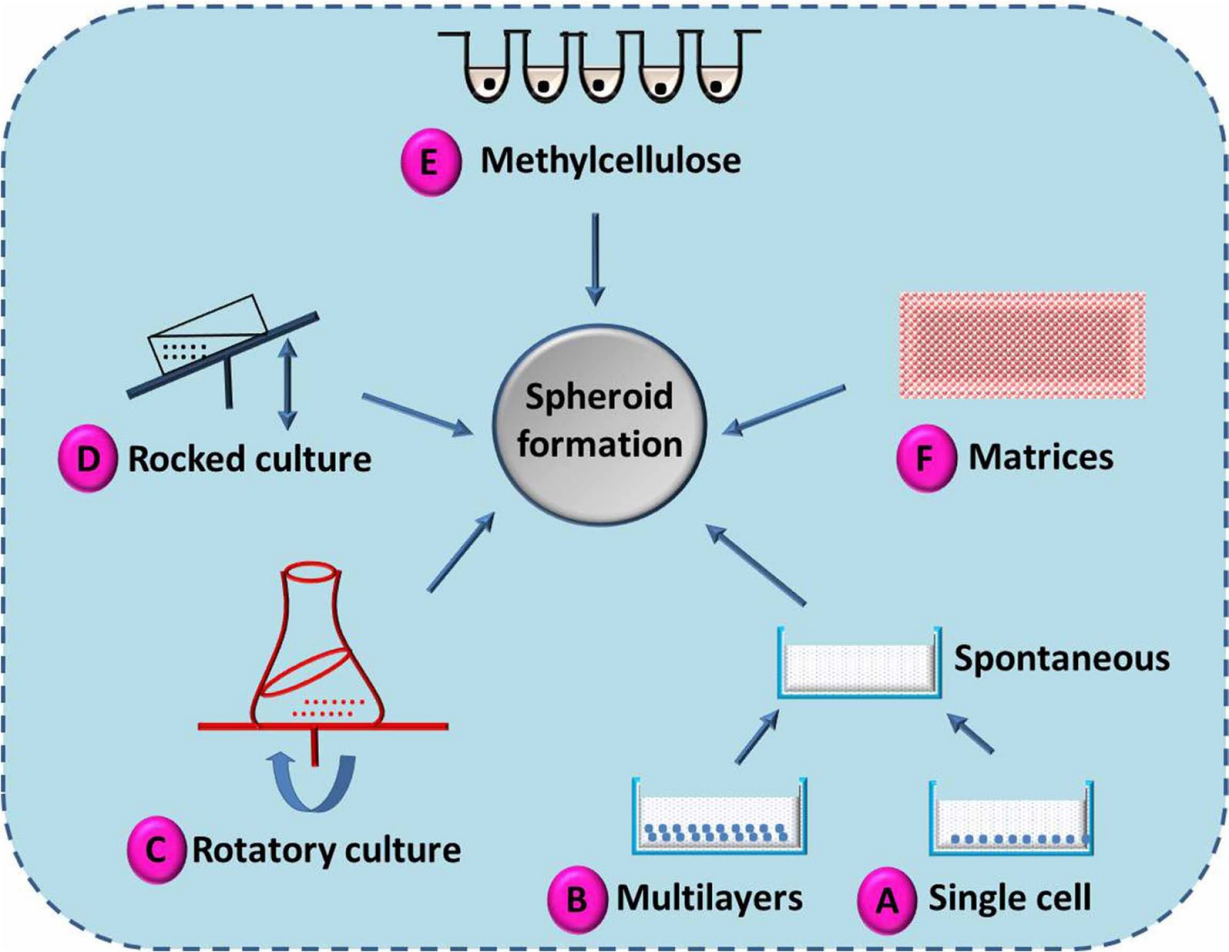
Various approaches for the development of 3D spheroid systems

The aggregation of cells into a spheroid structure provides the advantage of minimizing the exposed cell surface area [64, 80], with marked mimicking of natural cell organogenesis and morphogenesis [81]. Hence, the development of 3D spheroid cultures may be vital to investigate in vivo systems more efficiently, including cell morphology and the surrounding environment, which are reflected in the biological behaviour and gene expression of the cells. Several approaches are well established for the generation of 3D spheroid structures (Fig. 3).

Recently, a variety of 3D models have been developed to better understand the molecular and cellular interactions between various cell types with regard to HCC progression [82, 83]. In 2011, Tang and colleagues [84] established a novel 3D model for metastatic HCC by culturing MHCC97H cells on molecular scaffolds. Chemical, morphological, and pathological characterizations of the model showed several attributes that mirrored in vivo HCC, such as the morphological and ultrastructural features of neoplastic cells, gene expression patterns, apoptotic signals, glucose metabolism, and protein production. Additionally, xenografts of such 3D HCC spheroids in nude mice livers led to carcinogenesis and distant metastatic effects.

Another model of 3D multicellular hetero-spheroids was established by Yip and Cho [51] in a collagen hydrogel culture system to investigate the effect of TME, multicellularity, and the ECM barrier on the potential resistance of the tested anticancer drug. The uniform hetero-spheroid was formed with the hanging drop method and via co-culture of stromal fibroblasts with liver carcinoma followed by encapsulation in collagen gel to form a 3D spheroid. The results revealed that the chemotherapeutic resistance of the 3D hetero-spheroid model was higher than that of the homo-spheroid cultures and the 2D monolayer culture. In another study, Liu et al., [85] explored the relationship between HCC therapeutic resistance and 3D matrix stiffness using a model of alginate gel (ALG) beads with controlled matrix rigidity. They concluded that HCC cells in the ALG model with 105 kPa stiffness showed the highest therapeutic resistance against cisplatin, 5-FU, and paclitaxel. The authors suggested the role of endoplasmic reticulum stress-related genes in HCC drug resistance due to their upregulation in the 3D model compared to the 2D model.

In 2015, Terashima and colleagues [86] studied the expression of the drug-metabolizing enzymes CYP1A1 and CYP1A2 and their encoding genes in a 3D spheroid model of HCC cells (JHH1, Huh7, and HepG2). Results confirmed the increased expression of CYP1A1 and CYP1A2 in 3D spheroids compared to 2D cultured cells. As such, the authors concluded that the pregnane X receptor (PXR) increased CYP1A2 expression in JHH1, HepG2, and Huh7 spheroids. This study demonstrated the variation in gene expression between 3D spheroids and 2D cultured cells and between two different culture conditions.

Liu et al. [85] investigated the safety of in vitro co-culturing of umbilical cord mesenchymal stem cells (UCMSC), a common vehicle for anticancer drug delivery, on the growth and characteristics of cancer stem cells (CSCs) and on metastasis and therapeutic resistance of 3D-cultured HCC cells. The authors concluded that the metastatic ability of 3D HCC spheroids was greatly enhanced compared to the other cultures; this increase was confirmed to be due to upregulation of migration abilities, the expression of matrix metalloproteinases (MMPs), and EMT-related genes. However, the therapeutic resistance and cell growth of HCC cells were not affected. Moreover, the addition of the TGF-β receptor inhibitor SB431542 into 3D HCC cultures resulted in the reversion of EMT and downregulation of MMP-2 and migration ability.

Takai et al. [87] studied the biological features and signalling pathways of tumour cells in a 3D organoid-like spheroid model for HCC. They demonstrated that 3D spheroid cells could mimic the in vivo features of glandular epithelium and hepatic stem cells. Moreover, the authors showed the role of Wnt/β-catenin signalling activation in EpCAM + HCC spheroid formation and reported the chemotherapeutic resistance of EpCAM + HCC spheroids and their sensitivity to TGF-β-induced EMT.

Interestingly, Jung and colleagues [48] established a spheroid-forming unit to produce economic, large, and homogenous spheroids of liver neoplastic cells using Huh7 HCC cells. In these spheroids, proliferation and apoptotic signalling were present at the surface and centre of the spheroids, respectively, due to activation of ERK signal and hypoxia-induced factor-1 alpha (HIF-1α). Co-culturing of Huh7 HCC spheroids with 2% human umbilical vein endothelial cells (HUVECs) led to the expression of HCC-related genes, with a subsequent reduction in necrosis at the spheroid core and enhanced tumorigenic characteristics. Moreover, these 3D Huh7 cell spheroids showed increased therapeutic resistance against high concentrations of the chemotherapeutic drugs doxorubicin and sorafenib.

Recently, Sun et al. [88] developed novel 3D spheroid cell cultures by culturing HCCLM3 cells in 1% de-cellularized liver matrix-alginate (DLM–ALG) hybrid gel beads. They demonstrated that the DLM–ALG beads enhanced the activities of matrix MMPs, including MMP-2 and MMP-9, in HCCLM3 cells; a direct relationship was detected between MMP activities in HCCLM3 cells and the concentration of DLM powder used.

Another 3D HCC system was developed by Le et al. [89]; this model consisted of HCC cells, stromal cells in the form of fibroblasts, and nanofibrous membranes to imitate the complex TME. This model was fabricated by three methods of culturing: (1) a mono model in which tumour cells grow directly on the nanofibrous membrane, (2) a layer model in which fibroblasts grow on the nanofibrous membrane, and (3) a mixed model in which both tumour cells and fibroblasts develop on the nanofibrous membrane. Results showed that both the mono and layer models exhibited similar tissue features, while the mixed model resulted in phenotypic alterations of the neoplastic cells. In addition, the authors concluded that the mixed models enhanced the neoplastic cells’ resistance to chemotherapeutic drugs as well as the expression of vimentin and fibronectin.

Another 3D mixed-cell spheroid model using Huh-7 HCC cells and LX-2 stellate cells was recently developed by Khawar et al. [14] to mimic the natural in vivo TME and tumour-CAF interactions. The 3D system was cultured as mono-spheroids by culturing tumour cells alone or as mixed-cell spheroids in ultra-low attachment plates. ed Results showed enhanced type I collagen production and expression of pro‐fibrotic factors, including TGF-β1 and CTGF, compared to their levels in mono-spheroids. In addition, the expression of vimentin and E-cadherin was changed in the mixed-cell spheroids; these proteins promote the EMT phenotype. Drug sensitivity was enhanced in mixed-cell spheroids, and an anti-proliferative effect was shown only after combined treatment with sorafenib and oxaliplatin in a dose-dependent manner. Co-treatment with TGF-β inhibitors enhanced the therapeutic activity of sorafenib in the mixed-cell spheroids, suggesting the role of TGF-β in drug resistance.

Ma et al. [90] recently cultured HCC cell lines and fresh primary tumour cells in serum-free and ultra-low attachment conditions to enable the forming of HCC spheres and discovered that all cell lines and primary tumour cells shaped spheres. HCC spheres were capable of self-renewal, replication, and drug tolerance, as well as containing various subpopulations of CSCs. In immunocompromised animals, 500 sphere-forming Huh7 cells or 200 primary tumour cells could produce tumours. The shape of spheres was associated with tumour scale, numerous tumours, satellite lesions, and advanced stage. They came to the conclusion that there-forming culture would effectively enrich subpopulations with stem-cell properties, which are retained by activating the PPAR-SCD1 axis.

Xie et al. [91] developed a novel modelling method utilising three-dimensional (3D) bioprinting technology and constructed hepatorganoids with HepaRG cells, which maintain liver function and extend the survival of mice with liver failure after abdominal transplantation in an updated review. Following surgery, they collected HCC specimens from six people. Following that, patient-derived three-dimensional bio-printed HCC (3DP-HCC) models were successfully developed and grew well in long-term culture. These models maintained the characteristics of parental HCCs, such as stable biomarker expression, stable genetic mutations, and expression profiles. 3DP-HCC models are capable of showing drug screening outcomes both intuitively and quantitatively. They conclude that 3DP-HCC models are faithful in vitro models that can forecast patient-specific drugs for customised care and are accurate in long-term culture.

Overall, these results confirmed that different models of HCC 3D cell spheroids efficiently represent the physiological in vivo structure and function of HCC and the TME. Thus, these 3D models could be useful in advancing the study of tumour-stroma interactions as well as the molecular mechanisms underlying the therapeutic resistance of HCC.

## Conclusion

The development of an optimal experimental model is necessary to maximize the usefulness of preclinical investigations and to pave the way for creating and testing more novel, potential therapeutic drugs. Accordingly, obtaining in vitro models with high similarity to the natural in vivo conditions of HCC is key in future cancer studies. The use of 3D spheroid culture of HCC cells is promising for clarifying tumour-TME interactions and the mechanistic details of chemotherapeutic resistance, as well as for subsequently detecting more safe and effective anti-neoplastic drugs.

## Data Availability

All data generated or analysed during this study are included in this published article.
